# Socio-economic factors influencing the adoption of low carbon technologies under rice production systems in China

**DOI:** 10.1186/s13021-022-00218-6

**Published:** 2022-12-08

**Authors:** Zhong-Du Chen, Fu Chen

**Affiliations:** 1 China National Rice Research Institution, Hangzhou, 310006 China; 2 College of Agronomy and Biotechnology, China Agricultural University, No.2 Yuanmingyuan West Road, Haidian District, Beijing, 100193 China; 3 Key Laboratory of Farming System, Ministry of Agriculture of China, Beijing, China

**Keywords:** Farmer household, Climate change, Poisson estimators, Multivariate probit, Interdependent, Southern China

## Abstract

**Background:**

Rice (*Oryza sativa* L.) production, such as farmers’ livelihood and the soil quality, has been identified to be strong influenced by climate change in China. However, the benefits of low carbon technologies (LCTs) are still debatable in rice production for farmers, which have been identified to tackle agricultural challenges. The choice of potential LCTs relevant to the case study is based on a literature review of previous empirical studies. Thus, the objectives of the study were to (1) investigate the public perception and preferences of LCTs in rice production of China, and (2) analyze the influences of the factors on farmer’s decision in adopting LCTs in rice production. There were 555 farmer surveys from eight representative rice production counties in HP province of southern China, both the Poisson estimators and multivariate probit (MVP) approach were applied in the study.

**Results:**

Our results show that water-saving irrigation, integrated pest management techniques and planting green manure crops in winter season were the three major LCTs adapted by farmers in rice production. The intensity and probability of LCTs adoptions were influenced by the main factors including farmers’ education level, climate change awareness, machinery ownership, technical support and subsidies. There is a significant correlation among the LCTs, and the adoption of the technologies is interdependent, depicting either complementarities or substitutabilities between the practices.

**Conclusions:**

This study suggests that policies enhance the integration of LCTs would be central to farmers’ knowledge, environmental concerns, technical service and financial support in rice production systems in China.

## Background

Global climate change, associated with more extreme climate events, has been identified to increase the risks of floods, drought, and fire [1]. Agriculture is easily influenced by climate shifts, and predicted happened with relevant factors including redistribution of water availability and compromised quality, increased soil erosion, and decreased crop productivity [2, 3]. These factors present immediate and localized economic risks to farmers. In contrast, emissions of greenhouse gases (GHGs) pose potential threats to the larger landscape over a long time. Moreover, agriculture is the major source of the GHGs that are driving those changes, contributing about 53% and 78% of the total anthropogenic emissions of methane (CH_4_) and nitrous oxide (N_2_O), respectively [4]. With regard to CH_4_, rice (*Oryza sativa* L.) production remains the largest emission source from a single sector and accounts for 18% of total agricultural CH_4_ emissions [5]. Thus, climate change threats rice production systems, which represent negative effects to quality of life at local and global scales. What is more, as the economy develops and the population grows, increasing energy, chemical fertilizers, pesticides and agricultural films were instituted to maintain the grain yield, which further exacerbated the GHG emissions [5]. Therefore, development strategies of adaptation and mitigation for rice production systems is an urgent issue currently [2, 3, 6].

China is the largest rice producer in the world, accounting for 16% and 28% of the global rice area and global rice production, respectively [7]. The rice production is very important in China’s food security. However, rice production is very sensitive to climate change with the increasing of rice acreage during recent decades. The uneven spatial and temporal distribution of precipitation in the southern of China, especially during July and October when rice is in large water demand, high evaporation and low precipitation always lead to drought, in addition with poor irrigation infrastructure, which generally influenced the rice production. On the other hand, soils continue to deteriorate as a result of increased chemical fertilizer input, decreased organic fertilizer input, little application of green manure and soil erosion. According to a document released by Chinese government, the formulation and implementation of policies in adaptation to climate change have received high priority [8, 9]. In 2021, the governments of China had released a notification “Guidelines on accelerating the establishment and improvement of a green, low-carbon and circular economic system for development”, in order to build up low-carbon agriculture production systems. Low carbon rice technology can effectively reduce the energy consumption of mechanical operation, pesticide and chemical fertilizer application, and fix more organic carbon and nitrogen in the soil, which is not only conducive to reducing GHGs in paddy field, but also can promote the stable yield of rice. However, current knowledge about how to do farm management to implement these governmental plans is insufficient since previous studies were mostly either based on qualitative analysis or concentrated on other regions.

Generally, the optimized management strategies in agricultural systems have been identified to be useful to mitigate the GHG emissions, many of current applied technologies that can be implemented immediately [5, 10, 11]. However, most analysts were mainly concentrated on single technologies (e.g. nitrogen management, conservation tillage, or water-saving irrigation) adopted by farmers, which ignored the complementarities and/or substitutabilities of different technologies [12]. The extent of adoption of LCTs is measured by the number of component technologies adopted by rice farmers, which is more complex than the decision to adopt a single technology. The single decision is usually based on short-term profitability considerations, while interrelated adoption implies a more substantial and longer-lasting change in farming conservation [13, 14]. Moreover, technologies had been developed and disseminated as a package with several components by many scientists [15, 16]. Although previous studies have investigated the adoption of technology packages [17, 18], however, these studies are under the background of western countries where integrated management practices are usually adopted in dairy farming. Hence, the uniqueness evaluation of the package of technology is a major contribution of this study that had little studies on the adoption of LCTs investigation in rice farming in China. Therefore, the objectives of this study were (1) to investigate the application level of LCTs by farmers to cope with climate change in rice production of China; (2) to examine factors that affect the likelihood of farmers’ adoption intensity of selected LCTs by farmers in rice production; (3) to examine the effects of policy supports and household characteristics on farmers’ decisions in applying different LCTs in order to mitigate the effects of climate change, and considering the possibilities of adoption of different LCTs simultaneously.

## Methods

### Study areas

**Fig. 1:**
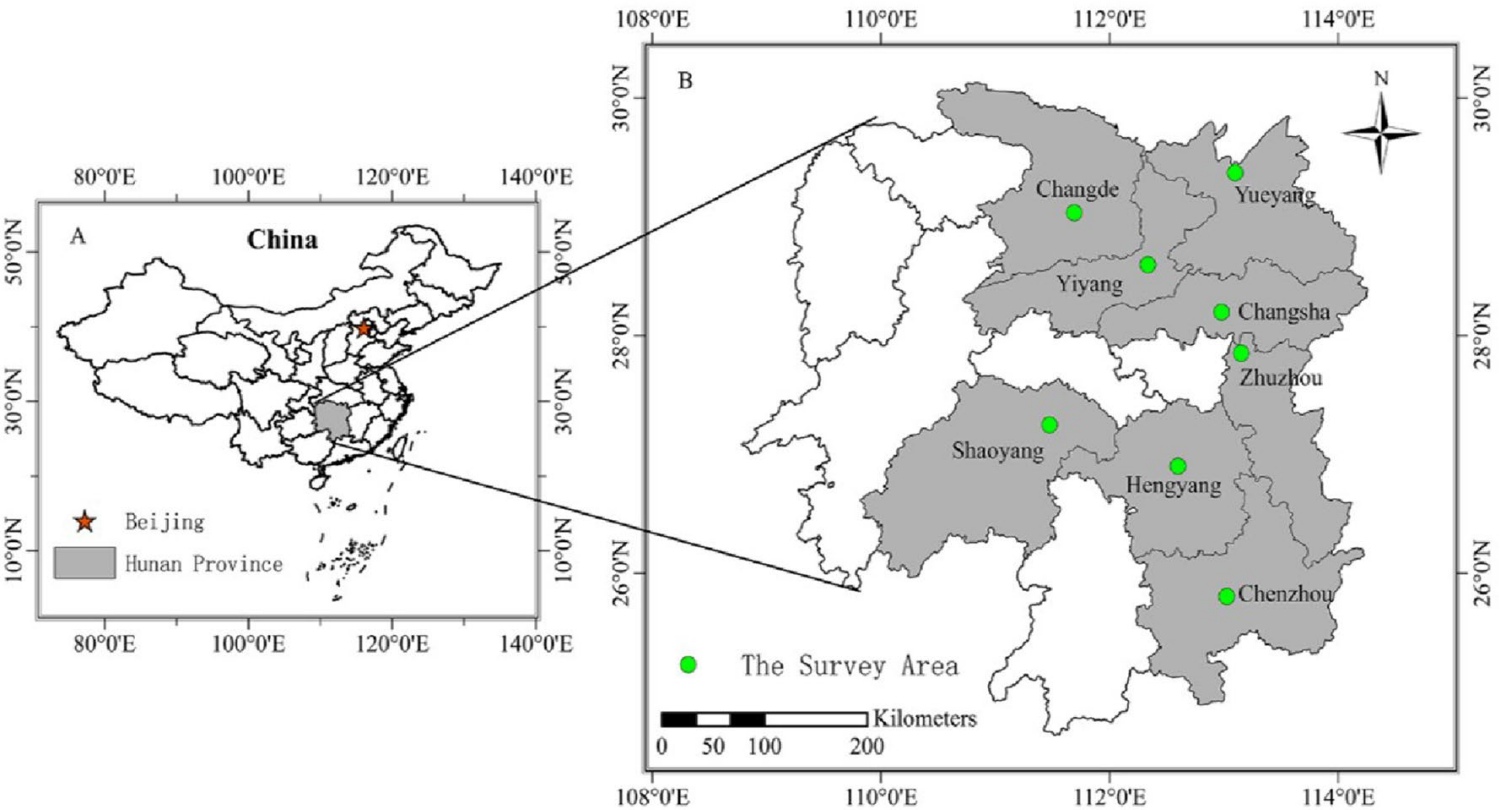
Map showing the location and distribution of the sampled holdings. **A** shows the location of Hunan in China. **B** further divides the region into its eight counties, from north to south, Changde, Yiyang, Yueyang, Changsha, Zhuzhou, Shaoyang, Hengyang and Chenzhou

The survey area of Hunan province (HP) selected typical provinces of rice production in China. HP is located in Southeast China (24°39′–30°08′N, 108°47′–114°15′E), which has a subtropical humid monsoon climate with an average annual air temperature of 16.4℃–18.5℃ which mean precipitation of 1200–1700 mm, 80% of which falls during the rice growing season from April to November. There are nearly 272–300 frost-free days and about 9 months with mean temperature above 10 g season from April to November. The province is one of major double rice cropping system provinces in China with 2.5 × 10^6^ ha double rice planting area in 2021. It was divided into three areas as follows: the northern commodity economy type, the central and eastern suburban type, and the southern export-oriented type. Eight representative counties were selected in this study, including Changde, Yiyang, Yueyang, Changsha, Zhuzhou, Shaoyang, Hengyang and Chenzhou (Fig. 1). The selection of the representative counties was based on the climate conditions, natural resources, soil fertility statue, socioeconomic conditions, geographic location and rice yield level amongst counties. The soil productivity statue was judged by the famers according to soil fertility, soil moisture content and topography in each field. Hengyang and Chenzhou in the south of HP represent the low fertility soil areas with low water resources, where the economic conditions are less developed. Changsha, Zhuzhou and Shaoyang in the central and eastern of HP represent the high fertility soil areas with high water resources, where the economic conditions are much better than other cities.

### Data sources

#### Selection of low carbon technologies for the case study

A review of agronomic experimental evidence in previous publications and studies shed some insight into discovering how LCTs help to reduce GHG emission. Retrieved from a keyword search of “mitigation/agriculture” in major scientific database platforms such as Web of Science, SciFinder Scholar and Google Scholar, previous studies that report successful agricultural practices in different regions that obtain higher mitigation potential in terms of soil carbon sequestration rate were collected. Table 1 shows the selection of these practices and the main sources of literature. The multiple benefits associated with the adoption of these practices have generated widespread social acceptance and scientific consensus. For example, conservation tillage is seen as a promising practice in increasing SOC stocks and reducing direct GHGs emissions [19, 20]. Moreover, Conservation tillage provides a large potential to offset indirect GHG emissions from energy and industrial sources through the reduced use of machinery [21]. Xu et al. [11] reported positive effects from reducing the time during which the soils are fully anaerobic though using intermittent drainage/irrigation or mid-season drainage for reducing CH_4_ emissions in flooded rice. Other studies found that benefits on GHGs reduction may be accentuated when using green manure due to more efficient nutrient use and reducing fertilizers application rate [11, 22]. Eighteen experts from research institutes and universities of regional and national levels were invited to evaluate and prioritize the practices identified in the above procedure with reference to socio-economic and environmental criteria. In choosing experts, the following guidelines were followed: (1) a minimum of 5 years’ working experience on issues related to GHG mitigation in agriculture; (2) sufficient knowledge of the different cropping management and systems so that the expert is able to cope successfully with the selected mitigation practices contained in the survey; (3) regular contact with farmers and extensive knowledge of the productive sector is prioritized. Apart from the survey of experts, the farmers were also asked to complete questionnaires containing selected practices from the literature review. The aim of the survey of farmers is to assess the current barriers to the adoption of the above practices in the case study area of HP. Though the survey of farmers also includes what other relevant mitigation measures adopted by them are, it gains no significant responses. The study of mitigation practices has revealed various options that could be applied in the present study.

**Table 1:** Detailed description of the seven selected low carbon technologies for this case study

Low carbon technologies	Description of the LCTs	Potential emission reduction rate	Sources
New rice varieties	Rice varieties, such as pest-resistant genetically modified varieties, efficient use of nitrogen fertilizer varieties, which can reduce the use of pesticides and nitrogen inputs or increase rice yield, or improve their oxidation in rhizosphere and transmission capacity, finally markedly reduce CH_4_ emissions	0.51–1.39 t CO_2_-eq ha^−1^	Tao, 2008; Fu et al. 2010; Xu et al. 2015
Conservation tillage	Reducing or avoiding tillage practices, which can increase soil carbon storage through reducing microbial decomposition, and promoting crop residue incorporation into soil	0.23–0.71 t CO_2_-eq ha^−1^	Zhang et al. 2013; Chen et al. 2014; Xue et al. 2014
Optimizing fertilizer management	Changes of fertilizer application rates, for instance, applying fertilizer depending on crop needs in different rice growth phases in order to increase fertilizer use efficiency thus reducing GHG emissions, especially nitrous oxide	0.36–0.62 t CO_2_-eq ha^−1^	Snyder et al. 2010; Shang et al. 2012; Chen et al. 2016
Water—saving irrigation strategy	This practice usually comprises one or several drainage periods in paddy soil, which prevents the development of soil reductive conditions and markedly reduces CH_4_ emissions	0.38–1.29 t CO_2_-eq ha^−1^	Ahn et al. 2014; Win et al. 2015; Xu et al. 2015
Pesticide reduction technology	It consists of reduced herbicide, hand weeding or pest control with light trap in order to reduce the pesticide inputs, thus reducing GHG emissions	0.48–1.85 t CO_2_-eq ha^−1^	Lei, 2013; Chen et al. 2016; Zhang et al. 2016
Planting green manure in fallow winter season	Planting green manure in the winter fallow field, increases soil carbon stores and reduced fertilizer use, thereby reducing nitrous oxide emissions	0.12–1.87 t CO_2_-eq ha^−1^	Xu et al., 2016; Shang et al. 2016; Wang et al. 2015
Planting-breeding technology	Common cultivation aquaculture in paddy fields, aerate the paddy soil by burrowing into the soil for searching food, prevent a drop in the redox potential and lower CH_4_ emission	0.78–2.12 t CO_2_-eq ha^−1^	Datta et al. 2009; Bhattacharyya et al. 2013; Xu et al. 2017

#### Questionnaire survey of low carbon technologies for the case study

The LCTs survey was a multiphase survey of rice farms in eight counties of HP in the present study. In terms of sampling, stratified random sampling was adopted with four parts of questionnaire including (1) overall information about the household and the household head; (2) farmer’s attitude towards climate change, low carbon agriculture and risk; (3) characteristics of the farmer’s filed; (4) external environment characteristics. The selection of variables has considered both economic theory and previous similar studies that conducting the adoption measures against climate change [23, 24]. There were 40 representatives were conducted as pre-tested at Swan village, Ningxiang county of HP in order to test the reasonability of the questionnaire. Finally, the questionnaire was efficiently improved based on the comments and suggestions. The reliability analysis was calculated by the Cronbach’s Alpha method, the results showed the Cronbach’s alpha coefficient were all over 0.7, which indicates that the data has good internal consistency of questionnaire and survey results have a high credibility. Finally, two townships in each county and two villages in each township for field surveys were randomly selected. Moreover, in each village, 20 farm households were randomly selected and interviewed. In the case of obtaining the electronic list of farmers, the method of generating random numbers is used. Assuming that the total number of farmers is n, we need to randomly generate 20 numbers between 1 and n by excel, and select the farmers in the order of these random numbers. If more than 3 households cannot participate in the survey, 20 random numbers will be produced again until less than 3 households cannot participate in the survey. The interviews were carried out among rice farmers during the period June–October 2013 and 2014. A 640 investigate dataset was collected from farmers across all eight counties. Ultimately, 555 surveys were finally used in the present study, which provided all information.

### Data and variable definition

Explanatory variables used in the econometric model and their expected signs are given in Table 2. Prior expectations about the relationships between the explanatory variables and the technology adoptions are based on theoretical underpinnings and from previous empirical results. On average, the age of rice farmers was around 50 years old, and rice farmers have approximately 6 years of formal schooling, 19 years of rice farming experience and 4 household members in HP. Farmers in this region have less on-rice income, accounted for approximately 25–49% of total revenue. The most of rice farmers in HP are more risk-averse, and lack of awareness of low-carbon agriculture. Each rice farmer has an average of 4 ha farm acreage, and very few rice farmers achieve farm mechanization, although they have a better supply of irrigation water in Hunan province. About 61% the rice farmers think their paddy soil is barren and unproductive. In addition, the famers in Hunan province find it difficult to obtain bank credit and technical support from government. It is notable that only 5% of the sample participated in on-farm demonstrations, and 10% of sample received training and technical assistance from government organization. About 61% rice farmers had achieved technology subsidies in this region.

**Table 2:** Statistical summary of dependent variables for the Poisson and the multivariate probit models

Variable	Description	Expected sign	Mean	SE
Dependent variable				
New rice varieties	Practice is implemented (1 = yes, 0 = no or not sure)		0.28	0.45
Conservation tillage	Practice is implemented (1 = yes, 0 = no or not sure)		0.17	0.38
Optimizing fertilizer management	Practice is implemented (1 = yes, 0 = no or not sure)		0.37	0.48
Water-saving irrigation	Practice is implemented (1 = yes, 0 = no or not sure)		0.57	0.49
Pesticide reduction technology	Practice is implemented (1 = yes, 0 = no or not sure)		0.50	0.50
Planting green manure in winter season	Practice is implemented (1 = yes, 0 = no or not sure)		0.46	0.50
Planting-breeding technology	Practice is implemented (1 = yes, 0 = no or not sure)		0.31	0.46
Low carbon technologies	Adoption intensity of low carbon technologies (taking on values from 0 to 7)		2.66	1.36
Independent Variable				
Farmer characteristics				
Gender	1 if the farmer is male; 0 otherwise	+	0.91	0.29
Age	Age of the farmers (years)	±	49.74	8.81
Education	Farmer having a formal education (no = 0, primary school = 6, junior high school = 9, senior high school = 12, university = 16)	+	6.39	4.01
Experience	Years of rice farming experience of the farmer	+	18.86	10.54
Household size	Number of family members	+	4.34	1.26
Farmer behavior				
Climate change awareness	1 if the farmer realize climate change; 0 otherwise	+	0.47	0.50
Low carbon agriculture awareness	1 if the farmer realize low carbon agriculture; 0 otherwise	+	0.33	0.47
Risk aversion	1 if the farmer practices crop diversification; 0 otherwise	+	0.35	0.48
Field characteristics				
Farm income ratio	Income ratio from rice farming of total income (1 = 0–24%; 2 = 25–49%; 3 = 50–74%; 4 = 75–100%)	−	2.41	1.12
Farm size	Total rice area planted in hectares	+	3.99	2.47
Machinery ownership	1 if the farmer owns any tractor or harvester; 0 otherwise	+	0.37	0.48
Soil fertility deficiency	1 if the farmer’s field is nutrient deficient; 0 otherwise	−	0.61	0.49
Sufficient water irrigation	1 if the farmer has an adequate source of water for irrigation; 0 otherwise	+	0.66	0.47
External environment				
Credit access	1 if the farmer has access to credit; 0 otherwise	+	0.44	0.50
Technical support	1 if the farmer get technical support; 0 otherwise	+	0.33	0.47
Subsidies	1 if farm subsidy received by implementing mitigation practices; 0 otherwise	+	0.61	0.49

The independent variables are the same across all models (n = 555)

“+” represents the expected positive effect, “−“ represents the expected negative effects, “ ± ” expected the impact uncertain

### Estimation of count data models

Low carbon technologies are characterized by a number of component technologies which can be adopted in sets by the farmer [25]. Thus, some farmers may adopt one or a few components, whereas others may adopt several or many components. The Poisson regression model can be considered the starting point for count data analysis, which was better used to predict the number of occurrences of the event of interest and the adoption of the selected LCTs in the present study. The dependent variable of the model (y) is a count of the number of LCTs adopted by farmers in a particular period; that is, y = 0, 1, 2, 3……, N. If y is a Poisson random variable, then its probability density function can be represented as [Eq. (1)]:

1 $$ f\left( {{\raise0.7ex\hbox{${y_{i} }$} \!\mathord{\left/ {\vphantom {{y_{i} } {{\text{x}}_{i} }}}\right.\kern-\nulldelimiterspace} \!\lower0.7ex\hbox{${{\text{x}}_{i} }$}}} \right)\, = \,{\text{P}}\,\left( {{\text{Y}}_{i} \, = {\text{y}}_{i} } \right)\, = \,\frac{{{\text{e}}^{\lambda } \lambda^{{\text{y}}} }}{{{\text{y}}^{!} }}\,\,\,\,\,\,{\text{y}}\,{ = }\,{0,1,2,3}..........{\text{,N}} $$

Where y_i_ is the number of LCTs adopted by farmer i and x_i_ are variables that affect the adoption of these practices. The factorial parameter y! is y factorial = y*(y − 1)*(y − 2)*2*1, whereas the expected mean parameter (λ) of this probability function is defined as follows:

2 $$ {\uplambda }_{{\text{i}}} \, = \,{\text{E}}\left[ {{{{\text{Y}}_{{\text{i}}} } \mathord{\left/ {\vphantom {{{\text{Y}}_{{\text{i}}} } {{\text{X}}_{{\text{i}}} }}} \right. \kern-\nulldelimiterspace} {{\text{X}}_{{\text{i}}} }}} \right]\, = \exp \left( {{\text{x}}_{{\text{i}}} } \right)\,\beta $$

The Poisson regression model is estimated by maximum likelihood. Some important conclusions are derived from the marginal effect concept, meaning that the change in the conditional mean of y when the regressors x change by one unit [Eq. (3)]:

3 $$\frac{\partial E\left[{y}_{i}|{x}_{i}\right]}{\partial {x}_{i}}$$

A negative binomial analysis as a statistical test has been carried out to allow an adjustment for the presence of over-under dispersion (variance of y_*i*_ greater or lower than its mean value) after running a Poisson regression. Over dispersion might mean that the regression experiences problems with inconsistency, deflated standard errors and grossly inflated t-statistics in the maximum likelihood output.

### Estimation of multivariate probit models

The multivariate probit (MVP) model was applied in this study to assess the multivariate adoption decision in the presence of adoption interdependence. It is a generalization of the probit model used to estimate several correlated binary outcomes jointly, which considers the possible contemporaneous correlation in the decision using different practices [26]. Furthermore, the MVP model can simultaneously estimate a variety of factors that affect the application of different technologies, and the relationship between the different technologies. Crucially, the fact that the decision of adopting a certain practice may be conditional on the adoption of another complementary practice (positive correlation in the error terms of adoption equations) or may be affected by the set of substitutes that are available (negative correlation, [27]). The observed outcome of LCT adoption can be modeled using a random utility formulation. Considering that the *h*^th^ farmer (*h* = 1, 2, 3 …, N) facing a decision to use or not to use the different LCT on a plot p (p = 1, 2, 3,…, p), U_0_ represents the benefit that the farmer uses traditional practices, and U_j_ denotes the benefit of using the *j*^th^ LCT: (j = S, T, N, W, M, F, P) that representing the adoption of new rice varieties (S), conservation tillage (T), optimizing fertilizer management (N), water-saving irrigation strategy (W), pesticide reduction technology (M), planting green manure in fallow winter season (F) and planting-breeding technology (P). When Y*_hpj_ = U_*j*_-U_0_ > 0, the *h*^th^ farmer will use the *j*^th^ LCT on plot p. Considering all LCTs, each equation in the system can be written as [Eq. (4)]:

4 $$ Y^{*}_{hpj} = \,X_{hpj} \beta \, + \,\varepsilon_{hpj} ,\,j = \,{\text{S, T, N, W, M, F, P}} $$

where Y*_hpj_ is a latent variable which can be represented by the level of expected benefit and/or utility derived from adoption, determined by observed household, plot and extension-related variables (X_hpj_) and unobserved characteristics (*ε*_*hpj*_), *β*_*j*_ is the corresponding vector of parameters [Eq. (5)]:

5 $$ {\text{Y}}_{{{\text{hpj}}}} = \,\left\{ {\begin{array}{*{20}c} 1 & {{\text{if}}\,\,{\text{Y}}^{*}_{{{\text{hjp}}^{ > 0} }} } \\ 0 & {{\text{otherwise}}} \\ \end{array} } \right. $$

where *Y*_*hpj*_ is the adoption of the *j*^*th*^ LCT by the *h*^*th*^ farmer on *p*^*th*^ plot. In the multivariate model, where the adoption of several LCTs is possible, the error terms have a multivariate normal (MVN) distribution with zero conditional mean and covariance matrix *W* with diagonal elements equal to unity (for identification of the parameters). The off-diagonal elements represent the unobserved correlation between the random components of the different LCTs. Thus, *ε*_*hpj*_ ~ MVN (0, *W*), and the covariance matrix *W* is given by [Eq. (6)]:

6 $$W=\left[\begin{array}{ccccccc}1& {p}_{st}& {p}_{sw}& {p}_{sn}& {p}_{sm}& {p}_{sf}& {p}_{sp}\\ {p}_{ts}& 1& {p}_{tw}& {p}_{tn}& {p}_{tm}& {p}_{tf}& {p}_{tp}\\ {p}_{ws}& {p}_{wt}& 1& {p}_{wn}& {p}_{wm}& {p}_{wf}& {p}_{wp}\\ {p}_{ns}& {p}_{nt}& {p}_{nw}& 1& {p}_{nm}& {p}_{nf}& {p}_{np}\\ {p}_{ms}& {p}_{mt}& {p}_{mw}& {p}_{mn}& 1& {p}_{mf}& {p}_{mp}\\ {p}_{fs}& {p}_{ft}& {p}_{fw}& {p}_{fn}& {p}_{fm}& 1& {p}_{fp}\\ {p}_{ps}& {p}_{pt}& {p}_{pw}& {p}_{pn}& {p}_{pm}& {p}_{pf}& 1\end{array}\right]$$

where $p$ (rho) denotes the pairwise correlation coefficient of the error terms corresponding to any two LCTs’ adoption equations to be estimated in the model. MVP is based on seven binary dependent variables, and each takes one if the farmer uses the respective practices during interview period in the 2013 cropping season, and zero otherwise. In this model, $p$ is not just a correlation coefficient, but carries more information. A positive correlation is interpreted as a complementary relationship, while a negative correlation is interpreted as being substitutes.

## Results

### Level of the adoption of low carbon technologies

**Fig. 2:**
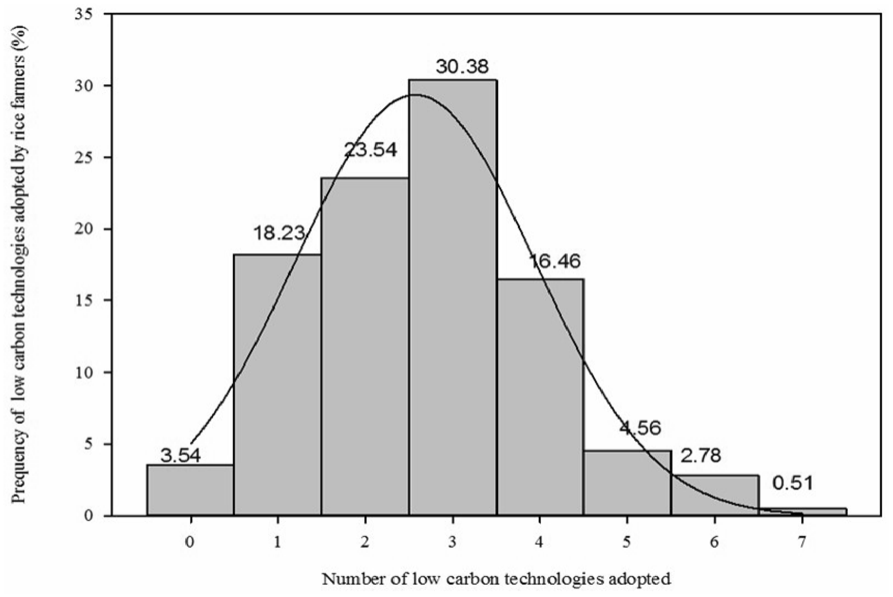
Distribution of total LCTs adopted by rice farmers in Hunan province of China

LCTs are a plat form that can be used to aggregate different technologies, which widely recognized as a key approach for the reduction of GHGs emission in rice-growing countries. Our results showed that the water-saving irrigation technology was the first adopted LCT by rice farmers, followed by pesticide reduction technology and planting green manure, respectively. In contrast, very few farmers adopted planting-breeding technology and new rice varieties in paddy soil. Lastly, minimum of LCT adoption by famers (18%) in rice production is conservation tillage. In respect of the distribution of the number of LCTs adopted by farmers (Fig. 2), the mean number of LCTs adopted by farmers is 2.66 with a standard deviation of 1.36. The distribution of farmers’ adopting LCTs showed normal distribution and slightly skewed to the left. About 30% of the farmers had adopted three LCTs, followed by choosing two LCTs in rice production. Around 18% of farmers adopted one or four kinds of LCTs, respectively. It also demonstrates that only 3 rice farmers adopt all seven practices (Fig. 2). The adoption of agricultural practices for GHG mitigation is a challenge for China farmers and farming advisers. Although the advisor’s knowledge related to sustainable soil management is very comprehensive, farmers’ attitudes and concern about GHG mitigation need further understanding in order to reach standardized practices that meet the new policy objectives.

### Determinants of the intensity of low carbon technologies adoption

A hypothesis test for over-dispersion (α = 0) was conducted to identify the most appropriate model. The log-likelihood values yielded by unrestricted negative binomial model are similar to the restricted Poisson model. The likelihood-ratio of 0.00001 is less than the χ2-critical value at 1% level of significance, which suggests the appropriateness of using the Poisson model. The result of the hypothesis test was confirmed by insignificant coefficient of the dispersion parameter. Therefore, the value, sign and significance of the estimated coefficients in these two models are identical to each other. Education was found to have a significant positive effect on the intensity of adoption of LCTs (Table 3). In addition, farmers who have big household size adopted more LCTs due to some of the LCTs (water-saving irrigation and planting-breeding technology) are labor intensive during rice growing seasons. However, these farmer characteristics have small marginal effects on the adoption of LCTs. In particular, more risk-averse farmers tend to adopt fewer LCTs, and farmers who had higher low carbon agriculture awareness applied more LCTs in rice production. The fact that increasing the awareness of climate change would lead to increased adoption of mitigation measures. The estimated marginal effects suggest that the increasing the awareness of climate change increases the likelihood of LCTs adoption by 27%. Furthermore, some of the crop practices in paddy soil such as conservation tillage, pesticide reduction technology and planting green manure require mechanical technologies for their large-scale implementation, thus it is not surprising machinery ownership significantly increased the intensity of LCTs adopted substantially. Technical support was also found to have a significant positive effect on the extent of LCTs adopted. In most cases, farmers need more technical support such as on farm demo or attendance at training to improve their capacity to apply the LCTs effectively. Thus, the availability acquirement of technical service will encourage the adoption of LCTs. Moreover, financial supports, like subsidies, were also presumed to be the determinants of the adoption of LCTs. Rational use of limited public budgets available for land use policies implies that subsidies are utilized to correct market failures, in order to incentivize recipients to make choices they would not do under market circumstances.

**Table 3:** Coefficient estimates and marginal effects of the Poisson regression model

Variable	Coefficient estimates		Marginal effects	
	Coef.	Std. error	Coef.	Std. error
Dependent variable: (number of LCTs adopted)				
Farmer and household characteristics				
Gender	0.0848	0.0720	0.2110	0.1787
Age	− 0.0180	0.0053^c^	− 0.0448	0.0131^c^
Education	0.0566	0.0175^c^	0.1409	0.0186^c^
Experience	0.0075	0.0055	0.0187	0.0137
Household size	0.0705	0.0396^a^	0.1754	0.0988^a^
Farmer behavior				
Climate change awareness	0.1109	0.0500^b^	0.2760	0.1250^b^
Low carbon agriculture awareness	0.0868	0.0306^b^	0.2161	0.0758^b^
Risk aversion	− 0.0264	0.0157^a^	− 0.0657	0.0391^a^
Field characteristics				
Farm income ratio	− 0.0327	0.0309	− 0.0814	0.0769
Farm size	− 0.0009	0.0127	− 0.0023	0.0317
Machinery ownership	0.0505	0.0160^c^	0.1258	0.0442^c^
Soil fertility deficiency	− 0.0398	0.0227^a^	− 0.0991	0.0567^a^
Sufficient water irrigation	0.0672	0.0465	0.1671	0.1153
External environment				
Credit access	0.0184	0.0058^c^	0.0459	0.0137^c^
Technical support	0.0590	0.0187^c^	0.1467	0.0414^c^
Subsidies	0.0110	0.0033^c^	0.0274	0.0075^c^
Constant	1.5385	0.4032^c^		
Log likelihood	− 829.021			
Prob. > chi2	0.00001			
Pseudo R^2^	0.1419			

Significant level of 10% (^a^), 5% (^b^) and 1% (^c^)

### Correlations among the adoptions of different low carbon technologies

Under the null hypothesis of multivariate probit models, the likelihood ratio test (chi^2^ (10) = 137.42, p = 0.00001) of the error terms are independent is strongly rejected. This statistical result shows that the error terms under the hypothesis in the LCTs adoption decision equations are correlated, and that under the MVP model is suitable in the case. Our results indicate that there is a significant relationship among LCTs, and the use of the practices is interdependent in that the probability of using a practice depends on the use of the other practices considered (Table 4). It is very vital to consider an alternative character among the different LCTs. Examination of these coefficients allows for the measurements of correlation between relevant LCTs adoption decisions after the influence of observed factors has been accounted for. The estimated correlation coefficients are statistically significant in five of the twenty-one cases, where two coefficients are positive and the rest three are negative. The positive signs of the correlation coefficients suggest that the decision to adopt one of the practices makes it more likely to use another practice. However, the different LCTs with negative signs of the correlation coefficients show that one of the practices has played supplementary role to another practice. For instance, there is a complementary effect between conservation tillage and optimized fertilization; moreover, planting green manure and optimizing fertilizer management also appear a complementary effect (Table 4). There is a substitution effect between water-saving irrigation strategy and pesticide reduction technology, pesticide reduction technology and planting-breeding technology, planting green manure and planting-breeding technology. The interrelation of the different LCTs sheds insight to the design of implementation strategies and polices in that a policy targeted on one of the LCTs could have spillover effect on the other practices.

**Table 4:** Correlation coefficients of LCT decisions: MVP model result

	S^1^	T	N	W	M	F
S						
T	− 0.118 (0.091)					
N	0.066 (0.089)	0.002 (0.092)^c^				
W	0.248 (0.082)	− 0.086 (0.083)	− 0.205 (0.081)			
M	0.005 (0.088)	0.085 (0.085)	− 0.014 (0.084)	− 0.109 (0.061)^a^		
F	− 0.129 (0.085)	− 0.111 (0.081)	0.065 (0.080)^c^	− 0.063 (0.076)	− 0.299 (0.272)	
P	− 0.042 (0.089)	− 0.199 (0.080)	− 0.134 (0.112)	− 0.258 (0.280)	− 0.170 (0.079) ^b^	− 0.323 (0.078)^c^
chi^2^(21)	137.419					
Prob	0.00001					
Likelihood ratio test		rho21 = rho31 = rho41 = rho51 = rho61 = rho71 = rho32 = rho42 = rho52 = rho62 = rho72 = rho43 = rho53 = rho63 = rho73 = rho54 = rho64 = rho74 = rho65 = rho75 = rho76 = 0				

S, T, N, W, M, F and P represent new rice varieties, conservation tillage, water-saving irrigation, optimizing fertilizer management, pesticide reduction technology, planting green manure in fallow winter season, and planting-breeding technology, respectively. The figure in bracket is standard deviation.

(^a^) indicates pearson correlation is significant at P < 0.1 level, (^b^) indicates pearson correlation is significant at P < 0.05 level, (^c^) indicates pearson correlation is significant at P < 0.01 level

### Determinants of individual low carbon technologies adoption

The MVP model provides a more detailed understanding of the factors influencing the adoption of individual LCTs in paddy field (Table 5). The hypothesis that the correlations between the error terms of the equations are all zero, and can be rejected at a high level of significance. This finding confirms that the MVP model fits the data better than the seven distinct univariate probit models (Table 5). Explanatory variables related to farmer characteristics had varied significantly across the seven dependent variables. Age of farmer was negatively associated with water-saving irrigation and planting green manure in fallow winter season. This is in line with our hypothesis that older farmers are less likely to adopt technologies which will drain physical strength largely. Higher education level was expected to encourage the adoption of complex and difficult LCTs such as conservation tillage, optimizing fertilizer management, integrated pest control technologies, planting green manure and planting combination management. The results presented here also indicated that the household size had a positive significant influence on the adoption of water-saving irrigation, integrated pest control technologies and planting green manure. The additional labor demands of the technology during labor scarcity have negative effects on the adoption of LCTs. Farming households whose family members are occupied with on-farm activities have a higher probability to adopt labor-intensive technologies.

**Table 5:** Multivariate probit results on the type of low carbon technology adoption

Explanatory variables	Dependent variables						
	S	T	N	W	M	F	P
Farmer and household characteristics							
Gender	0.280 (0.280)	0.268 (0.278)	0.245 (0.250)	0.014 (0.230)	0.223 (0.231)	0.036 (0.231)	− 0.013 (0.250)
Age	− 0.049 (0.018)	− 0.022 (0.019)	0.013 (0.017)	− 0.040 (0.017)^b^	− 0.007 (0.017)	− 0.037 (0.017)^b^	− 0.026 (0.017)
Education	− 0.035 (0.029)	0.113 (0.033)^c^	0.050 (0.028)^a^	0.039 (0.017) ^b^	0.140 (0.029)^c^	0.052 (0.028)^a^	0.058 (0.031)^a^
Experience	0.028 (0.020)	− 0.020 (0.021)	− 0.010 (0.019)	0.129 (0.177)	− 0.016 (0.019)	0.034 (0.018)	0.038 (0.019)
Household size	0.145 (0.155)	− 0.208 (0.159)	0.222 (0.149)	0.448 (0.146)^c^	0.369 (0.153)^b^	0.256 (0.144)^a^	0.103 (0.153)
Farmer behavior							
Climate change awareness	0.395 (0.205)	0.261 (0.109)^a^	0.512 (0.197)^a^	0.395 (0.118)^c^	0.223 (0.115)^a^	0.347 (0.203)^a^	0.639 (0.205)^c^
Low carbon agriculture awareness	0.258 (0.141)^a^	0.424 (0.135)^c^	0.042 (0.033)	0.186 (0.130)	0.016 (0.019)	0.195 (0.125)	0.237 (0.139)^a^
Risk aversion	0.057 (0.058)	− 0.096 (0.026)^c^	− 0.056 (0.058)	− 0.076 (0.054)	− 0.069 (0.066)	0.026 (0.056)	− 0.008 (0.060)
Field characteristics							
Farm income ratio	− 0.090 (0.076)	0.219 (0.143)	0.171 (0.140)	0.032 (0.119)	− 0.130 (0.127)	− 0.169 (0.131)	− 0.172 (0.139)
Farm size	0.055 (0.047)	− 0.062 (0.055)	− 0.063 (0.045)	0.015 (0.043)	− 0.073 (0.045)	− 0.007 (0.042)	− 0.001 (0.045)
Machinery ownership	− 0.066 (0.058)	0.710 (0.200)^c^	0.147 (0.175)	− 0.161 (0.174)	− 0.518 (0.179)^c^	− 0.113 (0.169)	0.765 (0.171)
Soil fertility deficiency	− 0.193 (0.102)^a^	− 0.098 (0.077)	0.153 (0.087)^a^	− 0.031 (0.089)	0.046 (0.092)	− 0.180 (0.087)^b^	0.020 (0.090)
Sufficient water irrigation	− 0.030 (0.025)	0.243 (0.072)^c^	0.109 (0.156)	− 0.388 (0.155)^b^	0.356 (0.163)^b^	0.044 (0.151)	− 0.095 (0.161)
External environment							
Credit access	0.075 (0.066)	− 0.081 (0.070)	− 0.008 (0.061)	− 0.097 (0.060)	0.219 (0.062)^c^	− 0.030 (0.059)^b^	0.144 (0.065)^b^
Technical support	0.425 (0.113)^c^	− 0.264 (0.073) ^c^	0.382 (0.158)^b^	0.366 (0.157)^b^	0.145 (0.166)	0.064 (0.153)	0.377 (0.164)^b^
Subsidies	0.067 (0.021)^c^	0.072 (0.022)^c^	0.072 (0.020)^c^	0.009 (0.018)	0.069 (0.020)^c^	0.043 (0.019)^a^	0.019 (0.029)
Constant	2.077 (1.074)^a^	− 1.073 (1.060)	− 0.978 (0.910)	0.969 (0.976)	− 1.134 (1.011)	1.792 (0.984)	1.317 (1.007)
Log likelihood	− 2034.61						
Prob. > chi^2^	0.0000						
Wald chi^2^ (112)	1165.23						

S, T, N, W, M, F and P represent new rice varieties, conservation tillage, water—saving irrigation, optimizing fertilizer management, pesticide reduction technology, planting green manure in fallow winter season, and planting-breeding technology, respectively. The figure in bracket is standard deviation.

(^a^) indicates pearson correlation is significant at P < 0.1 level, (^b^) indicates pearson correlation is significant at P < 0.05 level, (^c^) indicates pearson correlation is significant at P < 0.01 level

The second set of variables examines the relationship between farmers’ attitudes and adoption of technology. Our results showed that the farmers’ awareness of climate change has significantly positive impacts on the adoption of all LCPs apart from new rice varieties. This fits with our prediction that farmers would be more likely to adopt LCTs if they have a strong awareness of climate change. The next set of explanatory variables is composed of field characteristics factors such as farm income ratio, farm size, machinery ownership, and soil fertility and irrigation status. Machinery ownership has a positive significance test in the adoption of conservation tillage and pesticide reduction technology by farmers. Soil fertility deficiency was negative significant for new rice varieties and planting green manure. Farmers with fertile plots generally realize higher returns even without much investment in management, who were reluctant to invest in relatively costly inputs like drought or disease tolerant seeds, unless the productivity impacts are substantial.

The importance of financial and technical support has been highly recognized in the promoting the adoption of LCTs. Credit accesses for farmers to adopt LCTs are characterized by positive influence at pesticide reduction technology and planting-breeding technology. This suggested that farmers’ investment in the adoption of LCTs is affected by the financial institutions or government subsidies support, especially for some LCTs where a massive influx of funding is needed. However, credit-constrained households are more likely to adopt water-saving irrigation and planting green manure, both of which can be implemented by using household labor and thereby circumventing liquidity constraints. In our study, government technical service had a positive impact on the adoption of new rice varieties, through optimizing fertilizer management, water-saving irrigation and planting-breeding technology adoption by farmers. In fact, technical service for the LCTs from government has a significant positive effect on the adoption of LCTs using material objects (e.g., increasing organic fertilizer application, high-yield varieties, planting combination), while it plays an opposite role in the adoption of conservation tillage where farmers don’t have an intuitive appreciation for the effect of technology adoption. Subsidies were presumed to be the determinants of adoption decisions. These LCTs such as new rice varieties, conservation tillage, optimizing fertilizer management, pesticide reduction technology and planting green manure adoption by rice farmers have receives direct or indirect subsidies. The practices such as planting-breeding technology that do not receive subsidies may require a higher level of private investment and therefore their implementation relies only on the possible economic benefit for the farmer.

## Discussions

### Economic considerations of technology adoption by farmers

It is questionable whether policy makers should be directing farmers to treat their production activities as a farm business. But the decision to adopt is often an investment decision. And as Caswell et al. [27] noted, this decision presents a shift in farmers’ investment options. Smith et al. [28] pointed out that the economic limitations may be a huge barrier to the adoption of mitigation practices. Therefore, adoption can be expected to be dependent on cost of a technology and on whether farmers possess the required resources. Technologies that are capital-intensive are only affordable by wealthier farmers and hence the adoption of such technologies is limited to larger farmers who have the wealth [29]. In addition, changes that cost little are adopted more quickly than those requiring large expenditures; hence both extent and rate of adoption may be dependent on the cost of a technology. Economic theory suggests that a reduction in price of a good or service can result in more of it being demanded. Institutional factors deal with the extent or degree to which institutions impact on technology adoption by smallholders [23]. Researchers and development practitioners should be taken to avoid technologies with a high investment cost structure which smallholders cannot afford because they are poor and lack the necessary resources [23]. Considering that LCTs in our study can reduce resource consumption and environmental pollution while optimizing output, so the adoption of LCTs by micro farmers is an important way to achieve sustainable agricultural development. However, LCTs is not optimal for smallholder farmers who focus on short-term interests. How to change farmers preferences to adopt sustainable technologies that are both resource-saving and environmentally friendly has become the focus of scholars. Profit-oriented farmers are more attracted to the use of water-saving irrigation technology as the use of the technology will increase their utility [15]. Han (2011) [30] use Bivariate Probit econometric model to show that the adoption of soil testing and formula fertilization technology by farmers has a significant effect of increasing income. Conservation tillage can significantly increase crop yields [31]. Zhao [32] analyzed the impact of conservation tillage on yield and production cost, and concluded that the yield increase and cost saving effect of conservation tillage differed for some farmers. However, there are also some studies showing that conservation tillage has no significant impact on grain yield, and may even have a negative impact [33]. Wang and Zhang [34] confirmed that there is no significant correlation between the adoption of conservation tillage and the yield of wheat and maize based on the survey data of farmers in the Yellow River Basin of China, but it can significantly reduce the labor input. Instead, although conservation tillage will reduce human labor, it significantly reduces the success rate of seedling transplant, results in marked reductions in crop growth and grain yield [35]. What is more, research on integrated pest management technology has shown that it can help reduce the use of pesticides, while increasing yields, income and reducing costs.

### Factors of Farmers’ Decision to Adopt LCTS

Understanding farmer-specific characteristics and behavior as well as the production environment where farmers operate is an essential requirement before the dissemination of any rice technologies at the farm level. Baseline surveys are encouraged to characterize farmers so that the proper matching of rice technologies with farmer characteristics and agro-economic conditions on farms will reduce the cost of technology diffusion and mitigate the drawbacks of technology adoption. Because of the special system of China, subsidy becomes a practical way to improve the adoption probability of farmers. Since 2016, China has implemented a policy of subsidizing the system of arable land rotation and fallowing, piloting it in more than 10 major grain-producing provinces and regions. According to the corresponding standard, a subsidy of 320 $ ha^−1^ for crop rotation and 1070 to 1710 $ ha^−1^ for fallow land. Many researchers also found that farmers have an obvious advantage in the access to credit with larger farm sizes and capital, which determines its lower borrowing costs directly [12, 15]. Our study shows that subsidies were presumed to be the determinants of the adoption of LCTs. Rational use of limited public budgets available for land use policies implies that subsidies are utilized to correct market failures, in order to incentivize recipients to make choices they would not do under market circumstances [36].

Technical support was also found to have a significant positive effect on the extent of LCTs adopted. In most cases, farmers need more technical support such as on farm demo or attendance at training to improve their capacity to apply the LCTs effectively. Thus, the availability acquirement of technical service will encourage the adoption of LCTs. These results were in consistent with the previous studies [1, 37–39]. At present, some countries have established farmer school to guide farmers to master new technologies. Food and Agriculture Organization of the United Nations (2010) describe as an initiative to ‘build farmer capacity in entrepreneurial and management skills, via a ‘learning by doing’ approach’. The farmer school concept is based on continuous learning through experience that enables farmers to learn as they do things on their farm—what is commonly referred to as ‘action learning’. Management is combined with the technical aspects of agricultural production and marketing in a farmer school to influence the way that farmers make decisions and view the business world. Good extension programs and contacts with producers are a key aspect in technology dissemination and adoption. A publication stated that “a new technology is only as good as the mechanism of its dissemination” to farmers (Khanna [29]). Most studies analyzing this variable in the context of agricultural technology show its strong positive influence on adoption. In fact, Khanna et al. [29] show that its influence can counter balance the negative effect of lack of years of formal education in the overall decision to adopt some technologies. On the other hand, it also reflects the importance of the education level of farmers to the adoption of new technologies. The implementation of new practices is closely related to innovation of ideas and implementation by practitioners [40]. Age and education are essential determinants to innovation [41] and to agricultural innovation [40]. Higher education level was expected to encourage the adoption of complex and difficult LCTs such as conservation tillage, optimizing fertilizer management, integrated pest control technologies, planting green manure and planting combination management. The implementation of new practices is closely related to innovation of ideas and implementation by practitioners [40]. Age and education are essential determinants to innovation [41] and to agricultural innovation [40]. The estimated marginal effects presented here suggest that the increasing the awareness of climate change increases the likelihood of LCTs adoption by 27%. The fact that increasing the awareness of climate change would lead to increased adoption of mitigation measures is in line with many previous studies [42, 43]. The coefficient for “Risk aversion” was negative and significant for the farmer’s decision to adopt conservation tillage measures that were consistent with Bewket et al. [44] in northwestern highlands of Ethiopia.

### Correlation study of agricultural technology adoption

Among the many researches on the adoption of technology by farmers, special attention should be paid to the research on the integration of the adoption of technology by farmers. Mann [45] showed that agricultural technologies may consist of a series of different sub-technologies, and farmers will choose to use a combination of sub-technologies as needed in the agricultural production process, rather than using all sub-technologies, thus raising the issue of sub-technology package adoption for the first time. Feder [40] developed the theoretical model used in the technology package accordingly. Subsequently, scholars empirically explored the use of farmer sub-technology based on survey data in different regions. Rauniyar and Goode [46] empirically analyzed the use of seven sub-technologies by Swiss farmers, and found that farmers chose to use some sub-technologies among the seven sub-technologies, and three of the sub-technology combinations were most commonly adopted by farmers. Khanna [29] uses two specific techniques as examples, using a two-choice model to avoid sample selection bias, and empirically analyzing the common adoption of sub-techniques. Moyo and Veeman [47] demonstrated that farmers employ a set of technology bundles to achieve maximum utility when making technology adoption decisions. Yesuf and Köhlin [48] used a locally observable bivariate probit model to analyze the factors influencing farmers' fertilization and soil and water conservation technology adoption behavior. Our research comes to the same conclusion, suggesting that when farmers adopt LCTs, they do not adopt only one technology, but integrate technologies, the mean number of LCTs adopted by farmers is 2.66 with a standard deviation of 1.36 (Fig. 2). It is easily to understand that farmers are quite conservative facing the choice in the adoption of a new technology, which is more likely to adopt a mean number of new technologies [15]. Moreover, farmers decisions on technology adoption may not be independent, but simultaneous and interdependent, and studying a single technology in isolation ignores the economic information provided by the simultaneous use of multiple technologies and reduces the credibility of the research conclusions. A few scholars have begun to pay attention to the correlation effect between farmers adoption of sub-technologies in the technology package. Chu et al. [49] using the joint bivariate probit model to investigate the complementary effect between farmers application of commercial organic fertilizer and farm fertilizer. Wang and Huo [50] used the locally observable bivariate probit model to analyze and discuss the joint selection behavior of farmers orchard fine management technology. The results presented here also showed that there is a complementary effect between conservation tillage and optimized fertilization; moreover, planting green manure and optimizing fertilizer management also appear a complementary effect (Table 4). Gao et al. [51] found that long-term winter green manure incorporation significantly improved the paddy soil microbial properties and enzyme activities, which is an effective measure to improve the paddy soil health and fertility. There is a substitution effect between water-saving irrigation strategy and pesticide reduction technology, pesticide reduction technology and planting-breeding technology, planting green manure and planting-breeding technology. The interrelation of the different LCTs sheds insight to the design of implementation strategies and polices in that a policy targeted on one of the LCTs could have spillover effect on the other practices.

### Implications and Recommendations

As we know, the main sociodemographic determinant which affected farmers’ likelihood of adoption is the education level of farmers. The government extension service is an important factor that influences household’s adoption of LCTs that demand external knowledge and/or inputs. However, the government’s propaganda and support for LCTs are not strong enough although the government highlights the significance and necessity of climate change awareness. It is not enough to guide farmers to make technology choices. Technology subsidies for agriculture is important for their ability to deliver as an effective policy of technology adoption to climate change. These results would yield implications to help policy makers to design appropriate entry strategy to promote the use of LCTs. It is inappropriate to assert that smallholder farmers are reluctant to accept LCTs since different LCTs would require different entry points and promotion strategies. The result of the present study reveals that there is a significant correlation between the adoption of different LCTs and the use of the practices, which depicts either complementation or substitution among these practices. The potential correlation between the unobserved disturbances in the decision equations and the use of different practices could be ignored in the independent multiple-use decision model. The influence factors of estimates also produce deviation. The government should comprehensively consider the alternative and complementary effect of the farmer adoption decision in the agricultural technology promotion, and continue to improve the agricultural technology popularization system and strengthen the technical guidance such as implementation of the lecture field observation and field experiments. As expected, more educated farmers are in a better position to assess the relevance of new technologies [1, 52]. Meanwhile, we should also pay attention to the cooperative adoption of various farming technologies which exit a complementary relationship, and for the low carbon technology which exit substitutional relation, we need to consider actual circumstances, take measures to dispel the prejudice to the technology adoption, and encourage farmers to actively adopt various LCTs. Finally, a follow-up survey that screens out the adoption variables over time will enable researchers to conduct similar studies using a panel data set. This provides a more comprehensive analysis of farmers’ long-term adoption of new technologies.

## Conclusions

Our study applied the Poisson regression and MVP models to analyze the determinants of LCTs adoption by farmers in rice production. Our results showed that farmers have adopted various adaptation strategies to cope with global warming and increased extreme climate events in rice production. Water-saving irrigation, pesticide reduction technology and planting green manure in fallow winter season were the major adaptation strategies adopted by farmers in rice production. In addition, our results indicated that the factors influencing farmers’ adoption of LCTs are mainly affected by education, climate change awareness, low carbon agriculture awareness, household size, technical support and subsidies. Furthermore, farmers’ use of optimizing fertilizer management were packaged together with conservation tillage and planting green manure in fallow winter season. The substitution effect between integrated pest management techniques and planting-breeding technology adaption by famers can be found.

## Data Availability

The dataset supporting the conclusions of this article is included within the article.
